# Activation of AKT/PKB in breast cancer predicts a worse outcome among endocrine treated patients

**DOI:** 10.1038/sj.bjc.6600126

**Published:** 2002-02-12

**Authors:** G Pérez-Tenorio, O Stål, and members of the Southeast Sweden Breast Cancer Group

**Affiliations:** Department of Biomedicine and Surgery, Division of Oncology, Clinical Research Center, Faculty of Health Sciences, Linköping University, SE-581 85 Linköping, Sweden

**Keywords:** breast cancer, endocrine treatment, Akt, PKB, *erb*B-2, heregulin β1

## Abstract

Akt/PKB is a serine/threonine protein kinase that regulates cell cycle progression, apoptosis and growth factor mediated cell survival in association with tyrosine kinase receptors. The protein is a downstream effector of *erb*B-2 with implications in breast cancer progression and drug resistance *in vitro*. We aimed to examine the role of Akt-1 in breast cancer patients, by determining whether the expression (Akt-1) and/or activation (pAkt) were related to prognostic markers and survival. The expression of *erb*B-2, heregulin β1 and Bcl-2 was also assessed by flow cytometry or immunohistochemistry. This study comprised 93 patients, aged <50 who were treated with tamoxifen and/or goserelin. We found that pAkt was associated with lower S-phase fraction (*P*=0.001) and the presence of heregulin β1-expressing stromal cells (*P*=0.017). Neither Akt-1 nor pAkt was related with other factors. Tumour cells-derived heregulin β1 was found mainly in oestrogen receptor negative (*P*=0.026) and node negative (*P*=0.005) cases. Survival analysis revealed that pAkt positive patients were more prone to relapse with distant metastasis, independently of S-phase fraction and nodal status (multivariate analysis; *P*=0.004). The results suggest that activation of Akt may have prognostic relevance in breast cancer.

*British Journal of Cancer* (2002) **86**, 540–545. DOI: 10.1038/sj/bjc/6600126 www.bjcancer.com

© 2002 Cancer Research UK

## 

Endocrine therapy remains the most used systemic treatment in breast cancer. The presence of oestrogen receptor (ER) is the guide in the therapeutic decision. Complete oestrogen blockade seems to be more effective compared with single agent endocrine treatment. Combined therapy, involving luteinizing hormone-releasing hormone (LHRH) analogues such as goserelin (Zoladex) and tamoxifen has been employed to reduce oestrogen levels and to prevent the oestrogenic activity at the cellular level (Michaud and Buzdar, 2000). However in spite of higher response rate, tumour resistance to endocrine treatments develops over time and constitutes a critical problem (MacGregor and Jordan, 1998; Clarke *et al*, 2001).

Anti-oestrogen resistance may be explained by several mechanisms, including loss or mutation of ER, increased estradiol level, alterations in anti-oestrogen metabolism or interactions between growth factor receptors and ER cascades (reviewed in Clarke *et al*, 2001). These mechanisms, mainly involved in cell proliferation, may coexist with those affecting cell death. Increasing amount of evidence indicates that the mechanisms whereby drugs such as the LHRH analogues and tamoxifen exert the cytotoxic action also include apoptosis (Perry *et al*, 1995; Imai and Tamaya, 2000). Therefore, factors involved in the apoptotic failure may also contribute to the anti-oestrogen resistance.

Recently, the phosphatidylinositol 3′ kinase (PI3K)/Akt cascade, which is the major survival pathway for many cell types, has been related to activation of ER *in vitro* and with protection from tamoxifen-induced apoptosis (Campbell *et al*, 2001).

Akt, also called protein kinase B (PKB), is the cellular homologue of the vAkt oncogene found in the highly transforming retrovirus AKT8 (Ahmed *et al*, 1993). Human Akt exists as three closely related isoforms: Akt-1/PKBα, Akt-2/PKBβ and Akt-3/PKBγ. Akt-1 is activated by phosphorylation on two critical residues: threonine 308 in the kinase domain and serine 473 in the C-terminal tail. The active protein (pAkt) is a downstream effector of the growth factor-stimulated PI3K (Coffer *et al*, 1998) and may contribute to the genesis of cancer by blocking programmed cell death (Kandel and Hay, 1999).

From *in vitro* studies it is known that induction of cell survival by Akt isoforms affects signalling cascades triggered by a wide range of receptors. Platelet-derived growth factor (PDGF) (Klinghoffer *et al*, 1996), insulin growth factor (IGF) (Duan *et al*, 1999), and the epidermal growth factor (EGF) family members (Ram and Ethier, 1996; Okano *et al*, 2000) are among those receptors. Akt-1 has been found in correlation with higher expression of *erb*B-2 in a panel of breast cancer cell lines (Ahmad *et al*, 1999). A recent report showed that inhibition of this pathway with a monoclonal antibody against *erbB*-2 was not restricted to cells that overexpressed *erb*B-2 but also interfered with heregulin β1 (HRG)-mediated activation in cells with basal levels of the receptor (Liu *et al*, 1999). The former indicates that both, overexpression of erbB-2 and stimulation with HRG could be able to trigger this pathway (Tan *et al*, 1999; Ignatoski *et al*, 2000).

Until the present, Akt isoforms has been found overexpressed in several tumour types (Staal, 1987; Bellacosa *et al*, 1995; Cheng *et al*, 1996; Ruggeri *et al*, 1998) but the information in breast cancer is limited. In the present study we analyzed the expression and activation of Akt-1 in relation to some factors involved in its pathway as well as other tumour characteristics. We also aimed to examine the significance of this protein for patient's survival after endocrine treatment.

## MATERIALS AND METHODS

### Patients

This study comprized 93 breast cancer patients (35–49 years) from the Southeast Sweden Health Care Region who underwent surgery between 1984 and 1996. All patients were treated with tamoxifen, goserelin or both endocrine modalities. The tumours were positive for ER or progesterone receptor (PgR) in 96% of the cases. The medium follow-up time among the disease-free patients was 64 months. The first relapse appeared 20 months after surgery and further on 22 patients (24%) relapsed with distant metastasis. For this study we used frozen sections from tumours still available after hormone receptor assays. The material was stored at −70°C until used.

### Determination of steroid receptors and S-phase fraction

The levels of steroid hormones were measured using the EIA assay from Abbot (Lab. Diagnostic Division, Abbot Park, USA). Samples with ER or PgR ⩾0.3 fmol μg^−1^ DNA were considered as positive. Likewise, DNA flow cytometry was performed in clinical routine practice to determine the S-phase fraction (SPF). A cut-off level of 10% was used to differentiate tumours with low and high SPF respectively. Both methods have been described previously (Stål *et al*, 1992)

### Flow cytometry to determine* erb*B-2 content

This method was previously described (Stål *et al*, 1994). Fixation was carried out in 1% paraformaldehyde for 3 min. The cells were incubated with the monoclonal antibody c-*neu* (Ab-2), clone 9G6 (Oncogene Science Inc., Manhasset, NY, USA) or an irrelevant isotype control antibody (Sigma Chemical Co., St Louis, MI, USA), both diluted in PAB at 1 μg ml^−1^. The secondary antibody rabbit-anti-mouse F(ab)_2_ conjugated with FITC (DAKO, Denmark) was added at a 1 : 50 dilution. Finally the cells were treated with RNAse, stained with propidium iodide and analyzed in a FACScan flow cytometer (Becton Dickinson, USA). A fluorescence index (FI) was calculated as the ratio between the geometrical mean of the fluorescence associated with the specific antibody and the negative control. Tumours with FI ⩾2.0 were considered as positive.

### Immunohistochemical detection

The staining procedure followed a standard protocol. Imprints from nine tumours, otherwise frozen-sections (5 mm) were used for this purpose. Slides were fixed in acetone for 10 min at 4°C; blocked in PBS-10% swine serum (DAKO, Denmark) and incubated with the primary antibodies: a mouse monoclonal antibody, clone 124 (3.5 μg ml^−1^) (DAKO, Denmark) to detect Bcl-2; a sheep polyclonal antibody (at 8 μg ml^−1^) against the phosphorylated Ser residue in position 473 of human Akt-1(Upstate Biotechnology; Lake Placid, NY, USA); and goat polyclonal antibodies against-HRG (at 0.5 μg ml^−1^) and Akt-1 (at 8 μg ml^−1^) respectively (Santa Cruz Biotechnology, Inc). The negative control, in case of Bcl-2, consisted of a mouse IgG1 kappa-Mopc 21 (Sigma Aldrich, Sweden) at a concentration of 8 μg ml^−1^ while the polyclonal antibodies were incubated with their respective neutralizing peptides. Incubation with primary antibodies was performed overnight at 4°C in a moisture chamber. As secondary antibody a swine multi-link IgG1 anti-goat/mouse/rabbit conjugated with biotin and diluted 1 : 50 was employed, followed by streptavidin–horseradish peroxidase (DAKO, Denmark). Positive cells were visualized with 3.3-diaminobenzidin hydrochloride and the nuclei were counterstained with haematoxylin.

### Scoring

Two independent observers evaluated the sections using a light microscopy Leica DM LS (Leica Microsystems; Wetzlar, Germany). In case of HRG, we analyzed the staining associated to malignant cells and the surrounding stromal cells (fibroblasts). Tumours were considered positive for the stromal reaction when 10% or more stained fibroblasts were observed. In this case the imprints were excluded for being unreliable. In the malignant population we considered intensity (+ or ++) and frequency: (a) 0 cells; (b) 1–10% cells; (c) >10–50% cells; and (d) >50% of the cells being positive. The frequency and intensity were added to regroup the variable into three categories: (1) no reaction; (2) low reaction (1–10%/+/++ or >10–50%/+); (3) strong reaction (>10–50%/++ or staining in >50%/+/++).

For Akt-1 and pAkt, the tumours were considered positive, independently of the frequency, when the brown colour observed in the cytoplasm of the cells was strong and clearly different from that of the negative control. For Bcl-2, a cut-off point of ⩾10% was considered on the base of previous studies (Hellemans *et al*, 1995; Elledge *et al*, 1997).

### Peptide neutralization assay

Blockade with synthetic peptides tested the specificity of the polyclonal antibodies. The highest working dilution for the antibodies was established as 2 μg ml^−1^ in case of pAkt and 0.5 μg ml^−1^ in case of HRG. To be neutralized, the antibodies were pre-incubated overnight at 4°C with five and 10 times more peptide (for pAkt and HRG respectively) in a total volume of 100 μl and centrifuged at 10 000 *g*. Supernatants were applied to the sections.

### Statistics

The relationships between different variables were assessed by the Chi-square test or Chi-square test for trend, when required. The role of each variable (univariate analysis) or their joint effect (multivariate analysis) was evaluated with Cox's proportional hazard regression. The multiple sample test, which is a generalization of Gehan's generalized Wilcoxon test, was also used to test the significance in the survival analysis. All the procedures are comprised in the statistical package ‘Statistica’ (Statsoft, inc 1999 Statistica for Windows). The criterion for statistical significance was *P*<0.05.

## RESULTS

### Association of Akt-1 and pAkt with other variables

**Figure 1:**
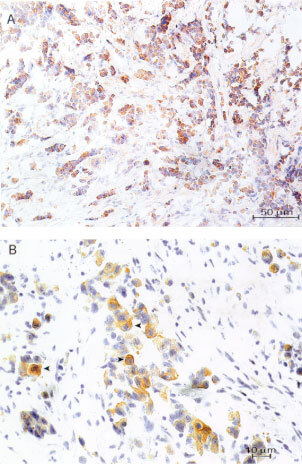
Representative immunostaining of Akt-1 (**A**) (200×) and pAkt (**B**) (400×), note the arrowheads indicating positive staining in the cytoplasm of malignant cells.

The pattern of staining for Akt-1 is shown in Figure 1A

. The protein was expressed in 46% of the tumours without significant correlation with any prognostic factor. On the other hand, pAkt, positive in 54% of the cases (Figure 1B), was positively correlated with the presence of HRG-expressing fibroblasts (*P*=0.017) and inversely to the SPF (*P*=0.001). No association was seen with nodal status, tumour size, ER, PgR, Bcl-2 or *erb*B-2 ( Table 1

**Table 1:**
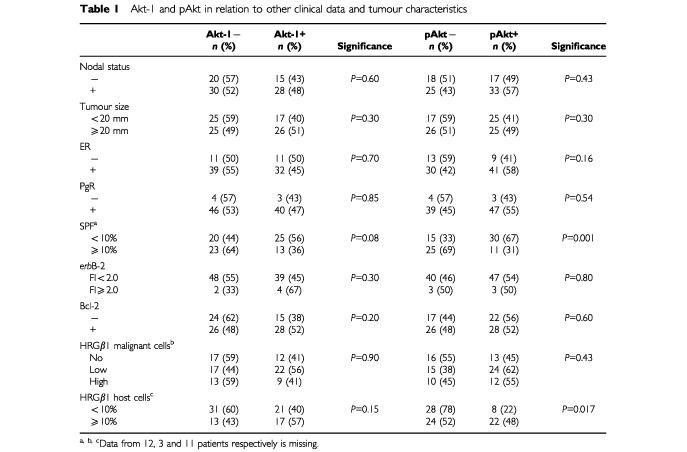
Akt-1 and pAkt in relation to other clinical data and tumour characteristics

). A moderate correlation, although statistically significant (*P*=0.04), was found between Akt-1 and pAkt staining. Besides, HRG, observed in malignant cells, was negatively associated with lymph node status (*P*=0.005) and with ER (*P*=0.026). The controls consisting of peptide-blocked antibodies were negative in all cases (result not shown).

### pAkt and other variables in relation to distant recurrence-free survival

**Figure 2:**
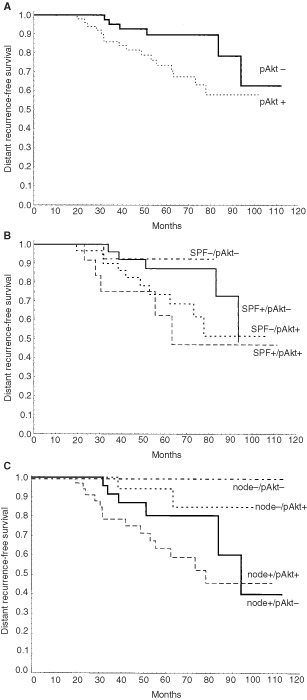
(**A**) Kaplan–Meier curves comparing survival among pAkt negative patients in comparison with pAkt positive group (*P*=0.07 by Cox proportional hazard model and *P*=0.03 by Gehan's generalized Wilcoxon test). (**B**) pAkt/SPF subgroups. The combined variable was scored as: 0 (SPF-/pAkt-); 1 (SPF+/pAkt-); 2 (SPF-/pAkt+); 3 (SPF+/pAkt+). According to this order, the risk of developing distant recurrence increased gradually with a rate ratio of 1.8 (Cox proportional hazard regression, *P*=0.017). Distant recurrence-free survival rates at 6 years were of 93, 88, 69 and 47% respectively for each subgroup. (**C**) Kaplan–Meier curves based on pAkt and nodal status. The combined variable was scored as: 0 (node-/pAkt-); 1 (node-/pAkt+); 2 (node+/pAkt-; 3 (node+/pAkt+). According to this order, the risk of developing distant recurrence increased gradually with a rate ratio of 2.5 (Cox proportional hazard regression *P*=0.001). Distant recurrence-free survival rates at 6 years were 100, 85, 81 and 59% respectively for each subgroup. In the lymph node negative group there were two recurrences; both belonged to pAkt+ subgroup.

In univariate analysis neither HRG nor Akt-1 had prognostic value, while pAkt positivity predicted a higher risk of developing metastasis with borderline significance (RR=2.4, 95% CI, 1.0–6.2) (see Figure 2A

). Multivariate analysis, including pAkt and traditional prognostic factors, indicated that pAkt was an independent predictor of distant recurrence ( Table 2

**Table 2:**
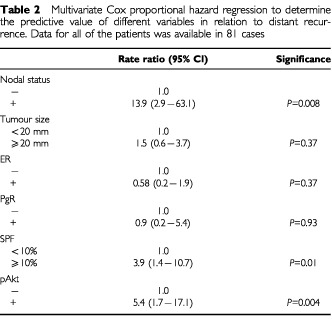
Multivariate Cox proportional hazard regression to determine the predictive value of different variables in relation to distant recurrence. Data for all of the patients was available in 81 cases

). The risk in the pAkt+ group was higher (5.4) compared to the pAkt− group; (*P*=0.004). Besides, the risk also increased significantly for the lymphonode positive patients (*P*=0.0008) and for those with high SPF (*P*=0.01).

The possible phenotypes of pAkt in combination with SPF are shown in Figure 2B. Patients with low SPF and pAkt negative tumour had a 6 year survival rate of 93% in comparison with 47% for those with high SPF and pAkt positive phenotype. The distant recurrence-free survival in relation to pAkt and nodal status is shown in Figure 2C. All patients in the double negative group (node-/pAkt-) remained alive after 6 years of follow-up while in the node+/pAkt+ group the distant recurrence-free survival rate fell to 59%.

## DISCUSSION

As far as we know this is the first immunohistochemical study analyzing the expression and activation of Akt in breast tumours in relation to other clinico–pathological variables and survival. We aimed to examine whether this protein, found *in vitro* to play a prominent role in oncogenesis (Kandel and Hay, 1999) and possibly in tamoxifen resistance (Campbell *et al*, 2001), showed the same significance in clinical material.

In this study, Akt-1 was considered positive in 46% of the tumours but it was not associated with any other prognostic marker. The variable did not provide any new information regarding the survival of the patients. Despite a positive correlation between Akt-1 and the activated protein, some Akt-1 negative cases resulted as pAkt positive. This paradox may be explained by either different sensitivity of the antibodies or by crossreactivity of the phospho-Akt-1 (ser473) antibody with another activated isoform present in the tumour, like Akt-2 or Akt-3. Otherwise, the pattern of staining with both antibodies was similar, being the phosphorylated protein localized to some extent to the cell membrane and mainly observed in the cytoplasm of the malignant cells. Albeit the activation of Akt is generally associated with its translocation from the cytosol to the plasma membrane by virtue of its pleckstrin homology (PH) domain (Andjelkovic *et al*, 1997), it is possible for Akt lacking a PH domain to be activated without being localized to this cell compartment (Sable *et al*, 1998).

As in case of Akt-1, we could not find any significant association of pAkt with nodal status, tumour size, ER, PgR or Bcl-2 but we did find a strong negative association with SPF. Since the activation of Akt promotes cell cycle progression by modulating the expression (Brennan *et al*, 1997; Muise-Helmericks *et al*, 1998) and stabilization of cyclin D1 (Graff *et al*, 2000) and the SPF is an indicator of the proliferative state of the cells, the negative association between pAkt and SPF was unexpected. Nevertheless, an increase in cyclin D1 does not always lead to cell cycle progression (Hiyama *et al*, 1997; Mitsuuchi *et al*, 2000), and in some cases the expression of cyclin D1 has been associated with low proliferative rate in clinical material (Nielsen *et al*, 1999). Furthermore, the survival analysis indicated that patients in the pAkt positive group tended to have a higher risk to develop distant recurrence compared to the negative group, and the multivariate analysis showed that pAkt was an independent prognostic factor in addition to nodal status and SPF. The analysis of the interaction between pAkt and SPF revealed that a low SPF was a favourable feature only if the tumour was in addition pAkt negative. This suggests that pAkt could be indicative of distant relapses in spite of a low proliferative state of the tumour, probably by promoting cell survival rather than cell proliferation.

In a previous report, including 20 adenocarcinomas, a significant association was found between the expression of *erb*B-2 and pAkt (Zhou *et al*, 2000). In the present study we found no significant association of *erb*B-2 with Akt-1 or with pAkt. We consider that our study could be undersized to detect significant differences, since only 7% of the tumours overexpressed the receptor (*n*=6) as expected in a sample conformed mostly by ER or PgR positive tumours. Therefore a larger material might help to confirm this relationship but also it may be interesting to address this question in the context of other EGF family members such as *erb*B-3, 4 and EGFR.

In this study we also explored the ligand HRG. The protein can be present in the cytoplasm of both malignant and stromal cells. While in a previous report, the host-derived HRG correlated with aggressive clinical behaviour (Visscher *et al*, 1997) we did not find the same result in our study. However we found a positive correlation between host-derived HRG and pAkt. It is known that the breast stroma plays an important role in breast epithelial growth and differentiation. Besides, tumour-associated fibroblasts have been found to confer morphogenic and mitogenic induction of epithelial cells (Shekhar *et al*, 2001) and to induce acceleration of epithelial tumour growth *in vivo* (Camps *et al*, 1990) supporting the role of host cells as source of paracrine signals that may not only affect cell growth but also turn on survival pathways, like Akt.

On the other hand, in those tumours where HRG was found in the malignant cells the patients less often showed evidence of lymphonodal spread in line with the results of Bacus *et al* (1994). The negative association found with ER status still could implicate this factor with an aggressive phenotype, although the risk to develop distant metastasis was not higher among the patients with high expression of HRG. A possible interaction with nodal status is not excluded since among the node positive patients the recurrence rate tended to be higher in cases of high HRG expression (result not shown). Nevertheless the role of HRG *in vivo* remains to be explained. This factor has been associated *in vitro* with induction of cell proliferation (Holmes *et al*, 1992; Aguilar *et al*, 1999), invasion (Hijazi *et al*, 2000), apoptosis (Daly *et al*, 1997), differentiation (Peles *et al*, 1992; Bacus *et al*, 1993) and inhibition of cell proliferation (Hamburger and Yoo, 1997; Xu *et al*, 1997).

We conclude that the activation of Akt could be a factor to consider together with S-phase fraction and nodal status in predicting distant relapses of breast cancer. However it remains to be elucidated which isoform plays the principal role. The shorter disease-free survival associated with the pAkt positive phenotype in this series of endocrine treated patients, may indicate a link between this pathway and treatment failure but further studies based on randomized trials are needed to validate this hypothesis.
